# Supplementary figures and images for: Ex vivo perfusion-based engraftment of genetically engineered cell sensors into transplantable organs
Source: PLoS One. 2019 Dec 2;14(12):e0225222. doi: 10.1371/journal.pone.0225222 (PMC6886851; doi:10.1371/journal.pone.0225222)

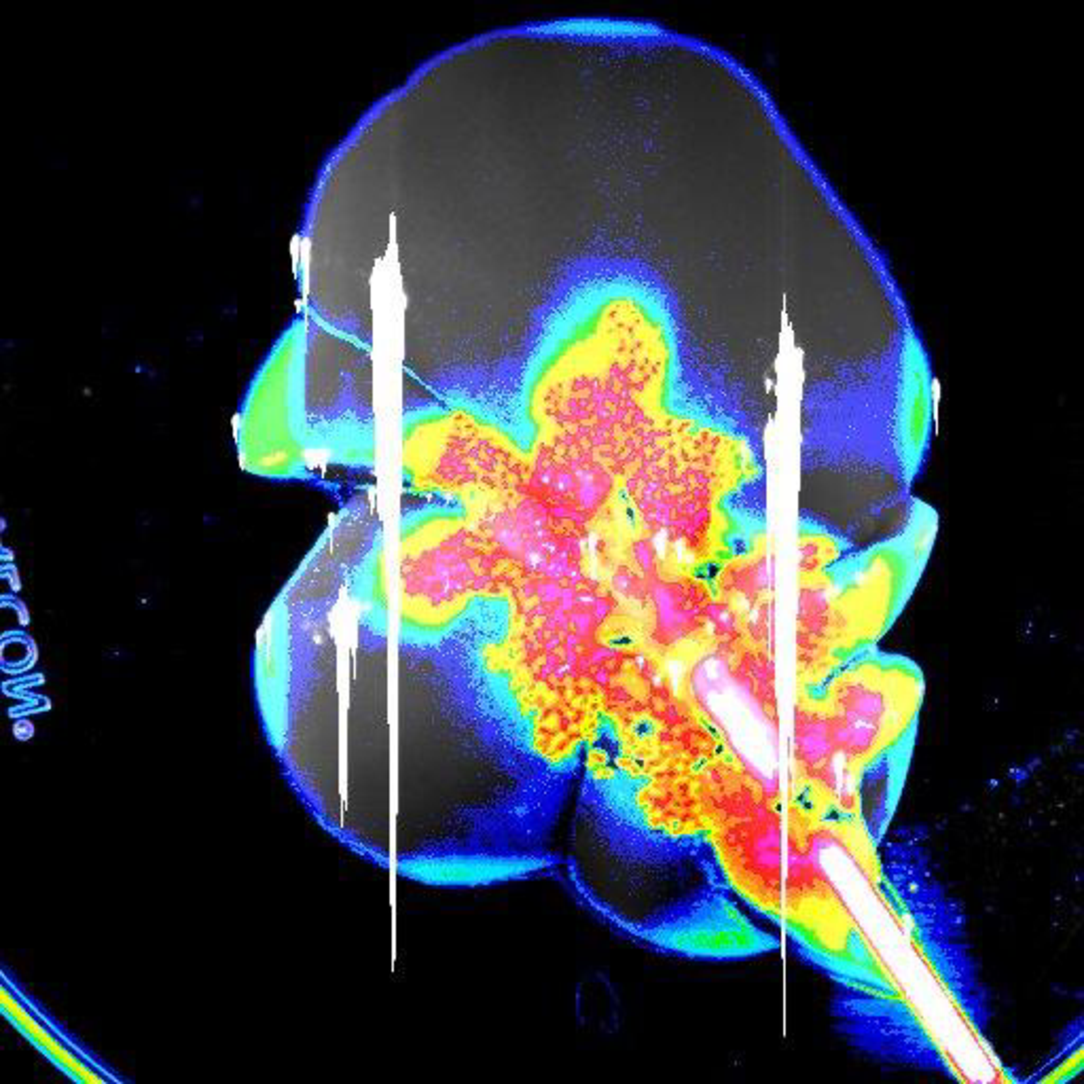

Supplement: S1 Fig — Dyed cells injected directly into the cannulated lived did not distribute as well as perfused cells. (TIF) [file pone.0225222.s001.tif]
