# Supplementary material for: Ex vivo perfusion-based engraftment of genetically engineered cell sensors into transplantable organs
Source: PLoS One. 2019 Dec 2;14(12):e0225222. doi: 10.1371/journal.pone.0225222 (PMC6886851; doi:10.1371/journal.pone.0225222)
Supplement: Attachment — Submitted filename: Response to Reviewer.docx [file pone.0225222.s002.docx]

Reviewer Comments

*Reviewer #1: In the study entitled “Ex Vivo Perfusion-Based Engraftment of Genetically Engineered Cell Sensors into Transplantable Organs”, Parekkadan et al. proves an innovative concept of integrating synthetic reporter cells ex vivo into organs as a transplant-within-a-transplant during functional organ preservation with a vision to use cell biosensors as a broad way to monitor and treat tissue transplants. Overall, this work has a degree of novelty and may be used for clinical application in future. However, I have a few comments as following:*

*1. The authors concluded that the method of Ex Vivo Perfusion-Based Engraftment in this study could be used for therapeutic application. However, they only tested the feasibility of Ex Vivo Perfusion-Based Engraftment using fibroblast cell model. As far as we know, a body of evidence shows that stem cells can display beneficial therapeutic effects on the rejection of liver transplantation and it is not hard to isolated stem cells from allogeneic sources. Therefore, in this regard I suggest that stem cell model should be tested at least.*

**Response.** We appreciate the comment and agree with the reviewer. Indeed, a recent study has already shown that ([PMC6347512](https://www.ncbi.nlm.nih.gov/pmc/articles/PMC6347512/)) Mesenchymal stem cells can be used therapeutically, in a paired human kidney perfusion study. We expanded the discussion on the use of stem cells in the revised manuscript accordingly. Our purpose in this study was to test if genetically-engineered cells can indeed be infused safely, without eliciting injury to the graft; careful selection of the cell type to be used requires evaluation of safety, persistence in vivo, sourcing and other concerns which will require further and thorough optimization for each cell type individually. The following language was added as an example to respond to our review:

“MSCs have also been demonstrated as a therapeutic adjuvant in perfused human kidneys [[PMC6347512](https://www.ncbi.nlm.nih.gov/pmc/articles/PMC6347512/)] to induce a regeneration response; it is therefore possible to envision future approaches where such innate therapeutic capabilities are combined with sensor functions engineered into the cells. For this initial study, we did not choose MSCs because such existing therapeutic effects would be possible confounding factors when analyzing the viability and function of livers during perfusion.

This study develops the protocols to introduce the engineered fibroblasts into perfused grafts, and demonstrates the cells cause no immediate injury or functional impairment to the livers, and their engineered functions remain stable during 6 hours of perfusion. However long term transplant studies are necessary to develop optimized protocols to ensure safety and function of	biosensor cells in vivo. Such studies would also test the long-term fate of cells, and compare other cell types with engraftment potential into a liver, such as endothelial cells, immune cells, or even hepatocytes themselves as natural considerations. We expect that the in vivo durability and fate of engineered cells could vary significantly between different cell types, and it is also possible that different applications will require different cell types. Other considerations could include additional	layers of safety, such as suicide switches that our team and others have	developed which can also be incorporated into biosensors for simple elimination after transplantation.”

*2. In fact, the mechanisms underlying the therapeutic effects on the rejection of liver transplantation are not fully elucidated. In many studies stem cells have been reported to function by secreting some key factors and exosomes, so it seems there may be no need for stem cells to dwell or distribute specifically in liver and then display protective effects after liver transplantation. Therefore, I suggest the authors to compare the therapeutic effects of stem cells between traditional injection method and Ex Vivo Perfusion-Based Engraftment method using rat liver transplant model.*

**Response.** We agree, however our aim is not to leverage innate therapeutic ability of stem cells, but rather to create novel, engineered cells that can function as sentinel cells which will be delivered to the grafts during perfusion-preservation. Dual-purpose engineered stem cells that have both therapeutic benefits and are engineered to perform as sensors is a valuable suggestion, and we revised the discussion section accordingly.

*Reviewer #2: Parekkadan, et al., present an interesting proof of concept study, wherein they demonstrate the ability of a machine-based perfusion system to distribute transducer fibroblasts, which produce Luciferase, into rat livers as demonstration that a system using in vivo biosensors might someday be used in the solid organ transplant settings to detect clinical events.

The overall concept is innovative and “out of the box.” They demonstrate in this article the ability to take fibroblasts, transduced with lentiviral vectors, and demonstrate that they successfully distribute throughout these organs. However, a number of questions that are relevant to the demonstration of proof of concept and practical ability to stimulate future translation, are worth considering.*

*First, one critical piece of this is demonstration that the distributed cells do not impair hepatic function. It is not clear to me that the demonstrations here are sufficient. For example, there is minimal edema and AST levels did not rise significantly, but the ideal demonstration of this would have been transplantation of a cellular biosensor-infused liver into an allogeneic (or even syngeneic) recipient with demonstration that the recipient animal can sustain adequate hepatic function.*

**Response.** We appreciate the Reviewer’s kind comments. We considered in the initial optimization stages a model that would provide faster results was appropriate. Selection of the metrics to assess liver injury (AST, oxygen uptake, lactate, glucose, weight gain and flow rates) is informed by literature in perfusion field where the same measurements are used clinically (PMC6659387) as well as our past work in developing indicators of viability in similar liver perfusion protocols (PMC3020900, PMC3441584). Specifically in this paper we used the viability criteria outlined by Mergental et al (PMC6659387), which uses a composite score that the livers show at least one major criteria (lactate clearance and/or bile production), and any two of minor criteria (stable arterial and portal flows, perfusate pH, and favorable macroscopic assessment by the transplant surgeon), and the livers with engineered cells meet these. The rationale in selection of these has been clarified in the revised manuscript. We agree that the transplant model is the next study to establish safety, as well as test selection of cell types to ensure long-term cell engraftment as also noted above and have briefly commented in the discussion section. The following language and data is provided:

“To assess viability of the perfused grafts, we used the composite viability criterion of Mergental et al which were established to clinically predict primary nonfunction in normothermically perfused human livers. Briefly, one major and two minor criteria must be met in order to deem the liver clinically viable. As shown in Figure 4, perfused livers containing engineered sensor cells produce bile (**Fig 4A**), a major criterion; show stable flow rates (**Fig 4B**) and stable perfusate pH (**Fig. 4C**), two minor criteria. Therefore, it can be suggested that the cell infusion and engraftment would not have an impact on the ability of the liver to successfully survive and function after transplant.

To further assess if the cell infusion did affect function, we assessed several additional parameters during perfusion: Metabolic activity of the liver was additionally tested using glucose stability^23^ as well as oxygen consumption rate. Hepatocellular damage was further assessed using and aspartate transaminase (AST) release [29] which we have also shown to be correlated to transplant success in similar normothermic rat liver perfusions systems (PMC3020900). Finally, we measured liver weight before and after perfusion to evaluate if there was any edema”

**Figure 4. Transplant viability assessment for biosensor engrafted livers.** A) Total bile collected during perfusion. B) Portal flow rates. C) Venous pH obtained from perfusate.

*Second, the choice of fibroblasts (vs., as discussed frequently, mesenchymal stromal cells that have a relatively broad history in clinical transplant settings) might become problematic, with the potential to induce inflammation vs. other cell types. Also the choice of a vector system capable of propagating into daughter cells might raise concerns about inflammatory (or even neoplastic) potential in the real world setting.*

**Response.** We agree with the reviewer. Also considering comments from reviewer 1, we appreciate that we did not articulate our reasons for choosing fibroblasts in this proof of concept study, nor further considerations that would need to go towards developing this approach for clinical suitability. We have revised the manuscript, in particular the introduction and discussion sections, to remedy this issue.
